# Exercise Programs for Muscle Mass, Muscle Strength and Physical Performance in Older Adults with Sarcopenia: A Systematic Review and Meta-Analysis

**DOI:** 10.14336/AD.2019.1012

**Published:** 2020-07-23

**Authors:** Wangxiao Bao, Yun Sun, Tianfang Zhang, Liliang Zou, Xiaohong Wu, Daming Wang, Zuobing Chen

**Affiliations:** Department of Rehabilitation Medicine, First Affiliated Hospital, Zhejiang University School of Medicine, Hangzhou, China

**Keywords:** Sarcopenia, exercise, elderly, meta-analysis, muscle, physical

## Abstract

Sarcopenia is an age-related condition that is characterized by progressive and generalized loss of muscle mass and function. Exercise treatment has been the most commonly used intervention among elderly populations. We performed a systematic review and meta-analysis to evaluate the available literature related to the effects of exercise interventions/programs on muscle mass, muscle strength and physical performance in older adults with sarcopenia. We searched PubMed, EMBASE, MEDLINE and the Web of Science for randomized controlled trials and controlled clinical trials exploring exercise in older adults with sarcopenia published through July 2019 without any language restrictions. Pooled analyses were conducted using Review Manager 5.3, with standardized mean differences (SMDs) and fixed-effect models. A total of 3898 titles and abstracts were initially identified, and 22 studies (1041 individuals, 80.75% females, mean age ranged from 60.51 to 85.90 years) were included in the meta-analysis. The exercise programs in the studies consisted of 30 to 80 min of training, with 1 to 5 training sessions weekly for 6 to 36 weeks. Muscle strength (grip strength [SMD 0.57, 95 % CI 0.42 to 0.73, P <0.00001] and timed five chair stands [SMD -0.56, 95 % CI -0.85 to -0.28, P < 0.0001]) and physical performance (gait speed [SMD 0.44, 95 % CI 0.26 to 0.61, P < 0.00001] and the timed up and go test [SMD -0.97, 95 % CI -1.22 to -0.72, P < 0.00001]) showed significant improvement following exercise treatment, while no differences in muscle mass (ASM [SMD 0.15, 95 % CI -0.05 to 0.36, P = 0.15] and ASM/height^2^ [SMD 0.21, 95 % CI -0.05 to 0.48, P = 0.12]) were detected. Exercise programs showed overall significant positive effects on muscle strength and physical performance but not on muscle mass in sarcopenic older adults.

Sarcopenia was first described in the 1980s and has been recognized as an independent muscle disease; sarcopenia has had an ICD-10-MC Diagnosis Code since 2016 [1]. The European Working Group on Sarcopenia in Older People (EWGSOP) [2], the Asian Working Group on Sarcopenia (AWGS) [3] and the International Working Group on Sarcopenia (IWGS) [4] have provided a similar definition of sarcopenia with different cut-off values depending on study populations. Nowadays, sarcopenia is diagnosed as an age-related and progressive condition characterized by low muscle mass, muscle strength and physical performance and is associated with increased falls, fractures, frailty, and mortality [5, 6]. In 2019, the EWGSOP2 was published by Cruz-Jentoft, et al, updating the definition and highlighting that low muscle strength is a key characteristic [7].

Appendicular skeletal muscle mass (ASM), total body skeletal muscle mass (SMM) and muscle mass adjusted for body size (height squared, weight and body mass index) were used as indicators of muscle mass to assess sarcopenia [8]. For example, ASM <20 kg for men and <15 kg for women, ASM/height^2^ <7.0 kg/m^2^ for men and <6.0 kg/m^2^ for women were used as cut-off points for diagnosing sarcopenia according to the EWGSOP2. Dual-energy X-ray absorptiometry (DXA), bioelectrical impedance analysis (BIA), computed tomography (CT) and magnetic resonance imaging (MRI) are common techniques to measure muscle mass. In addition, grip strength and chair stand tests are simple and effective measurement in clinical practice [9, 10] and are routine methods to evaluate muscle strength to identify sarcopenia: grip strength <27 kg for men and <16 kg for women and chair stand test >15 s for five rises (as defined by the EWGSOP2). Finally, physical performance has been previously detected by gait speed and the timed up and go (TUG) test [11], contributing to the assessment of the severity and prognosis of sarcopenia among elderly individuals [12]: gait speed ≤0.8 m/s and TUG test ≥20 s (as defined by the EWGSOP2).

It is widely accepted that physical exercise, especially resistance training [13], is effective for improving muscle function and functional ability among older adults. Habitual exercise has been confirmed to be beneficial for preventing sarcopenia in elderly individuals regardless of the exercise type and intensity [14]. However, the implementation of exercise treatment for sarcopenia has just begun, and the correlation between exercise programs and sarcopenia-related symptoms remains unclear. Previous literature regarding exercise interventions suggested muscle strength and physical performance, even muscle mass have been increased in older adults with sarcopenia, whereas no consensus recommendations on physical exercise for the prevention of sarcopenia have been made due to the existence of multiple contributing variables [15]. Therefore, this study aims to perform a meta-analysis of randomized controlled trials and controlled clinical trials and systematically assess the effects of exercise programs on muscle mass, muscle strength and physical performance in older adults with sarcopenia.

## MATERIALS AND METHODS

### Data sources and search strategy

This systematic review and meta-analysis were performed according to the Preferred Reporting Items for Systematic Reviews and Meta-Analyses (PRISMA) standards [16]. The protocol of this study was registered at PROSPERO (Center for Reviews and Dissemination, University of York: CRD42019141658).

A systematic literature search for randomized controlled trials and controlled clinical trials published from January 1990 to July 2019 was conducted using PubMed, EMBASE, MEDLINE and the Web of Science. The search included the keywords ‘sarcopenia’, ‘sarcopenic’, ‘exercise’, ‘physical’ and ‘training’ (the PubMed search strategy, which was used for all the databases, is available in the supplementary files). The electronic search was then supplemented with a manual search of the bibliographies of the identified studies. No restrictions on the language of publication was applied during the database searches.

### Inclusion and exclusion criteria

The reference lists obtained were independently screened by two investigators (BWX and SY) in accordance with the inclusion and exclusion criteria, and disagreements regarding study eligibility were resolved by a third investigator (ZTF). After screening the titles and abstracts, the initially eligible articles were selected for a full text review.

Articles were included if they met all of the following criteria: 1) participants were diagnosed with sarcopenia based on any established definitions (by a working group, a certain article or clinical experience); 2) mean or median age ≥60 years; 3) physical exercise training was performed, without a limitation regarding exercise type; and 4) the assessment of muscle mass, muscle strength or physical performance was reported.

Studies were excluded if 1) no original data was included (review, protocol, abstract, etc.); 2) they were animal studies; 3) they were performed with young or middle-age populations; 4) the participants had other accompanying diseases (e.g., cancer, liver cirrhosis, diabetes, stroke, depressive disorder, and metabolic syndrome); 5) there was no comparison group; 6) no outcome of muscle mass, muscle strength or physical performance was included; and 7) the exercise intervention was combination with other interventions (nutrition)

### Data extraction and quality assessment

The following information was extracted: authors, year, number of participants, age, sex, body mass index (BMI), diagnostic criteria, training period, training frequency, exercise intensity or workload, exercise modality, program design, ASM, ASM/height^2^, SMM, SMM/height^2^, grip strength, five chair stand time, gait speed, TUG test and other pre/postintervention performance indicators.

**Table 1:** Characteristics of the included studies.

Refs	N		Age	Sex (F, %)	BMI	Diagnostic Criteria	Period	Weekly (times)	Intervention
	Exe	Con							
[27]	14	16	68.47±2.78	0.00%	23.37±1.91	ASM/Height^2^ < 10.75 kg.m^-2^	8w	3	10 min warm-up, 45 min resistance training.
[23]	8	8	84.30±5.37	58.93%	21.90±3.01	AWGS	12w	2	5 min warm-up, 20 min resistance exercise program and 5 min cool down.
[26]	40	37	73.39±6.92	75.32%	18.85±2.04	AWGS	24w	1	5-10 min warm up and cool down routine, 20-30 min chair-based resistance exercises using Thera-Bands, and 20-min aerobic exercises.
[33]	35	37	69.95±2.73	100.00%	24.80±0.91	EWGSOP	36w	2	5 min warm-up, 20 min muscular districts with low weight loads
[18]	33	31	79.90±7.80	50.00%	25.00±3.39	SMM/Weight < 37.15% for men and < 32.26% for women	12w	2	60-min warm-up, muscle resistance training and relaxation stage.
[25]	11	17	81.75±6.96	30.72%	31.28±6.44	SMM/Weight ≤ 0.93 for men and ≤ 0.57 for women	12w	3	5 min warm-up, 20-30 min resistance exercises with workload and 5 min cool down.
[34]	36	18	72.87±7.02	87.30%	22.72±2.45	EWGSOP	12w	2	60 min comprehensive progressive group exercise program or home therapeutic exercises
[17]	17	16	67.48±4.29	100.00%	N.A.	AWGS	8w	2	60 min progressive resistance training
[36]	33	23	67.35±5.23	100.00%	28.05±3.77	SMM/Weight < 27.6 %	12w	3	10 min warm up, 40 min elastic resistance exercises and 5 min cool down.
[22]	25	25	74.10±6.15	100.00%	27.30±1.74	SMM/Weight < 25.1 %	24w	5	50-80 min of combined aerobic and resistance exercises
[32]	25	21	67.32±5.20	100.00%	27.72±3.30	EWGSOP	12w	3	5 min warm up, 35-40 min resistance training exercises and cool down routine.
[24]	15	15	68.83±3.36	83.33%	27.83±3.80	SMM/Weight ≤ 32.5% for men and ≤25.7% for women	8w	2	60 min progressive resistance load training using weight-training equipment at 60-70% of one repetition maximum.
[35]	60	20	75.75±5.42	70.00%	23.84±3.01	ASM/Height^2^ < 8.87 kg.m^-2^ for men and 6.42 kg.m^-2^ for women	12w	3	Whole-body vibration training with 14,400 vertical vibrations
[31]	7	8	60.51±2.40	100.00%	19.47±1.52	EWGSOP	6w	2	60 min training program comprised five exercises (squats, front lunges, side lunges, calf raises, and toe raises).
[38]	18	17	69.20±4.94	100.00%	28.11±3.66	SMM/Weight < 27.6 %	12w	3	10 min warm up, 40 min elastic resistance exercises and 5 min cool down.
[37]	14	14	72.00±4.05	100.00%	32.50±2.62	Handgrip strength≤21 kg	10w	2	60 min progressive resistance exercise program
[21]	35	34	81.25±4.68	100.00%	25.20±2.63	SMM/Height^2^ < 5.67 kg.m^-2^	12w	2	60 min warm-up, weight/machine training, stationary bicycle aerobic exercise, and chair/standing exercise.
[29]	20	21	85.90±7.50	64.44%	29.09±4.52	EWGSOP	24w	2	60 min progressive resistance and balance training.
[30]	26	14	68.96±5.76	55.77%	20.09±1.81	AWGS	24w	varying	home exercise programs, combining walking with lower limb resistance exercises.
[28]	19	16	79.90±4.92	27.69%	24.92±3.55	ASM/Height^2^ < 10.75 kg.m^-2^ for men and 6.75 kg.m^-2^ for women	12w	2	10 min warm up, 30 min resistance exercises using Thera-Bands, and 10 min cool-down.
[20]	32	32	68.60±5.54	100.00%	N.A.	ASM/Height^2^ < 6.42 kg/m^2^	12w	2	60 min of stretching, muscle strengthening, balance and gait training.
[19]	39	39	78.85±2.84	100.00%	18.85±1.85	ASM/Height^2^ < 6.42 kg/m^2^	12w	2	5 min warm up, 30 min strengthening exercise, 20 min balance and gait training and 5 min cool down.

ASM= Appendicular Skeletal Muscle Mass; AWGS= Asian Working Group for Sarcopenia; BMI= Body Mass Index; EWGSOP= European Working Group on Sarcopenia in Older People; SMM= Skeletal Muscle Mass; SD= Standard Deviation; w=week; y=years.

**Figure 1.**
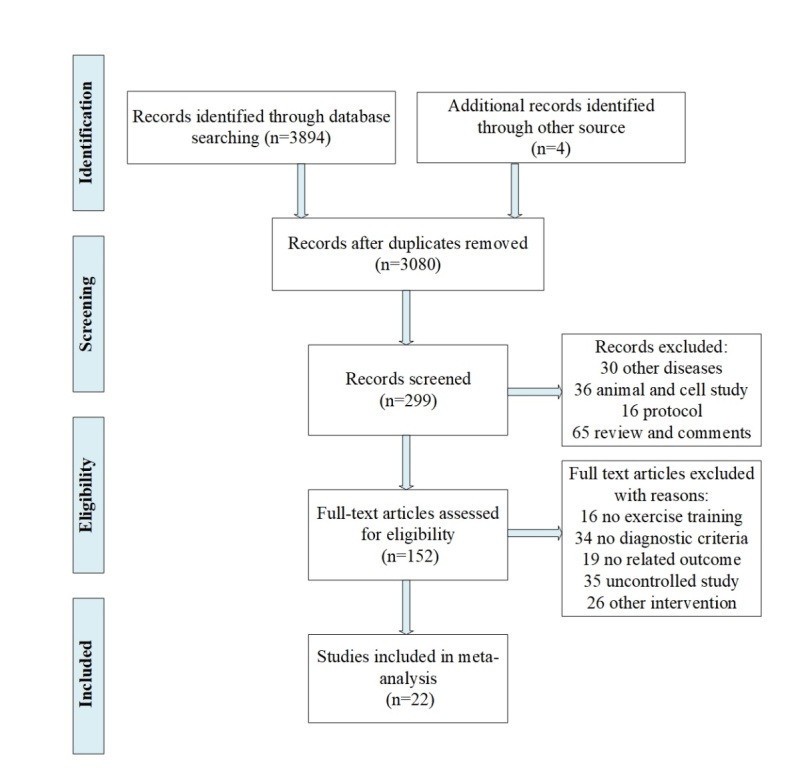
Flow diagram of studies search, selection and inclusion process.

The risk of bias of the included trials was assessed by two independent investigators (BWX and ZTF) using Review Manager 5.3 software (Cochrane Collaboration, UK), and disagreements regarding the methodological quality were resolved by discussion. The quality assessment was performed according to the Cochrane criteria, including selection bias, performance bias, detection bias, attrition bias, reporting bias, and other potential biases, which were categorized into three grades: low risk, unclear risk and high risk. Percentage of the three groups were then calculated.

### Outcomes and effect size calculation

All the outcomes were continuous variables, and pooled analyses were conducted using Review Manager 5.3, with standardized mean differences (SMDs) and fixed-effect models. The standardized effect sizes and 95% confidence intervals (CIs) were calculated to test the results. The degree of heterogeneity of the effect sizes was quantified with the I^2^ statistic, ranging from 0% to 100%. Possible sources of heterogeneity within the study were investigated using subgroup analyses stratified by different exercise programs. Further, a sensitivity analysis was conducted to determine the robustness of our results.

To assess the risk of publication bias, funnel plots and Egger’s test were conducted using StataSE V.13 (StataCorp, College Station, Texas, USA). A P value less than 0.05 was considered significant for all analyses.

## RESULTS

### Study characteristics

We identified 3894 titles and abstracts in the databases, and 4 records from study references were initially identified. After removing duplicates and screening the titles and abstracts, 152 full-text articles remained and were further assessed for eligibility. According to the inclusion and exclusion criteria, 19 randomized controlled trials [17, 19-28, 30, 32-38] and 3 controlled clinical trials [18, 28, 31] were finally included in the meta-analysis (Fig. 1).

**Figure 2.**
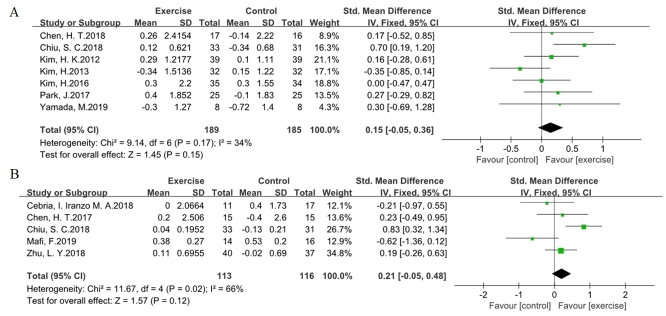
**Effects of exercise programs on the muscle mass in older adults with sarcopenia**. Forest plot of difference in mean change from baseline in ASM **(A)** and ASM/height^2^ **(B)** after the intervention. ASM, Appendicular skeletal muscle mass. CI confidence interval, IV, inverse variance, Std, standardized.

The characteristics of the included studies are shown in Table 1 [17-38]. The number of participants in each study ranged from 15 to 80, and the mean BMI ranged from 18.85 to 32.50 kg/m^2^; a total of 1041 older adults with sarcopenia were involved in the studies. The mean age of the participants ranged from 60.51 to 85.90 years, and 80.75% of participants were female. The exercise programs included resistance exercise, home-based exercise, aerobic exercises, power training, whole-body vibration training and combination training. The duration of the interventions ranged from 6 to 36 weeks, and the exercise sessions consisted of 30 to 80 min training.

**Figure 3.**
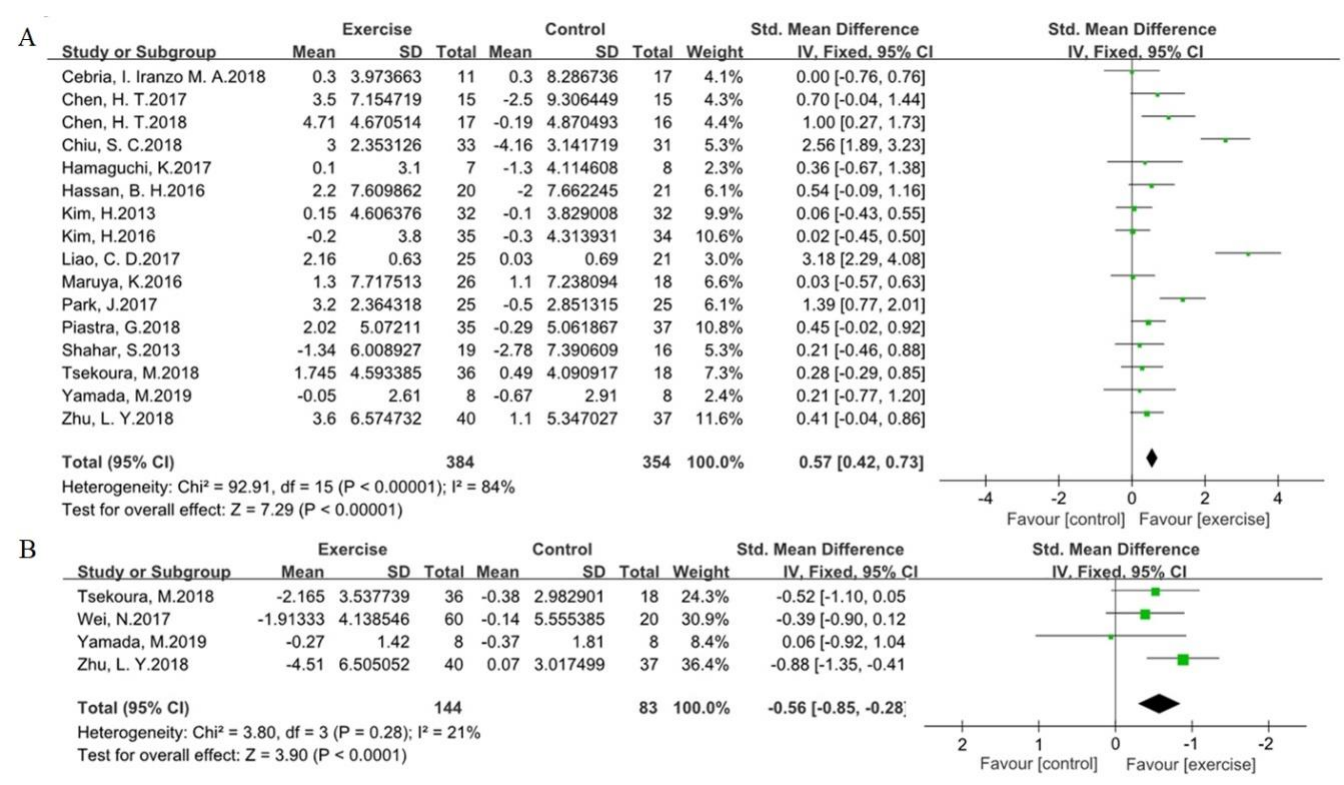
**Effects of exercise programs on the muscle strength in older adults with sarcopenia**. Forest plot of difference in mean change from baseline in grip strength **(A)** and five chair stands time **(B)** after the intervention. CI confidence interval, IV, inverse variance, Std, standardized.

**Figure 4.**
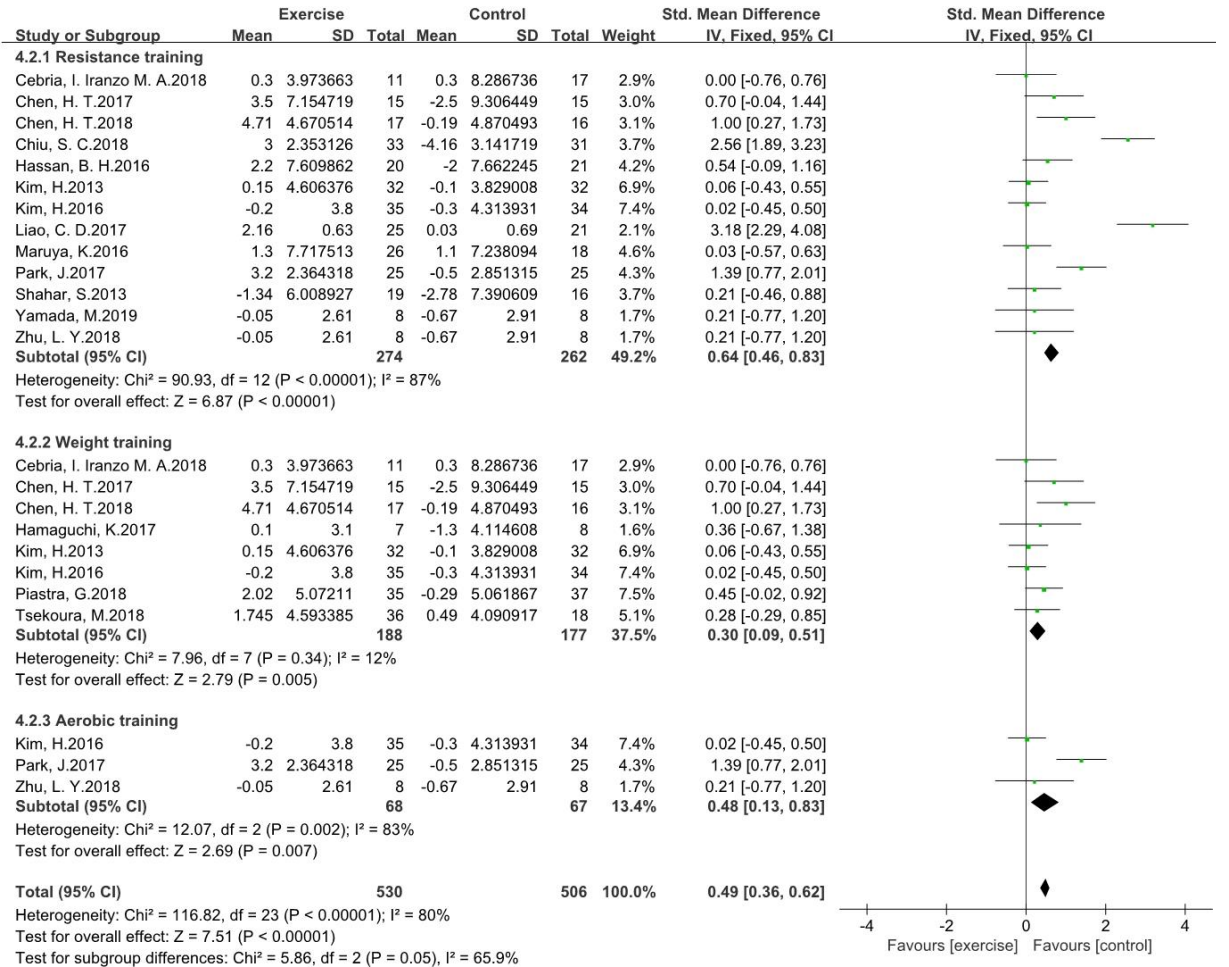
**Effects of different exercise programs on the grip strength in older adults with sarcopenia**. Forest Forest plot of difference in mean change from baseline for grip strength in sarcopenic individuals after **(A)** resistance training, **(B)** weight training, **(C)** aerobic training. CI confidence interval, IV, inverse variance, Std, standardized.

The included studies assessed muscle mass (ASM [17-23] and ASM/height^2^[18, 24-27]), muscle strength (grip strength[17, 18, 20-26, 28-33] and five chair stand time[23, 26, 34, 35]) or physical performance (gait speed[19-22, 25, 26, 29, 32, 34, 36, 37] and the TUG test[20, 27, 32, 34-36]) at baseline and after the exercise intervention. Huang [38] used skeletal muscle mass/weight as the outcome for muscle mass. In addition, three studies used SMM [17, 24, 33], and four studies used SMM/height^2^ to represent the muscle mass [30, 31, 34, 34].

### The effect on muscle mass

ASM and ASM/height^2^ were selected to evaluate the efficacy of the exercise program on muscle mass in older adults with sarcopenia (Fig. 2). Seven trials included information regarding ASM, and five trials included information regarding ASM/height^2^, which were pooled together in the method of inverse variance using a fixed-effect model. The value for the change in overall effect size in the general assessment indicated that no significant effect of exercise was shown for ASM (SMD 0.15, 95 % CI -0.05 to 0.36, P = 0.15, I^2^ = 34 %) or ASM/height^2^ (SMD 0.21, 95 % CI -0.05 to 0.48, P = 0.12, I^2^ = 66 %). In addition, the outcomes of SMM (SMD 0.21, 95 % CI -0.13 to 0.55, P = 0.23, I^2^ = 0 %) and SMM/height^2^ (SMD 0.29, 95 % CI -0.01 to 0.59, P = 0.06, I^2^ = 0 %) showed no significant difference (Supplementary Fig. 2).

### The effect on muscle strength

Grip strength and five chair stand time were selected to evaluate the efficacy of the exercise programs on muscle strength in older adults with sarcopenia (Fig. 3). Sixteen trials included information for grip strength, and four trials included information regarding five chair stand time, which were pooled together using the method of inverse variance with a fixed-effect model. The value for the change in overall effect size in the general assessment indicated that the efficacy of exercise was statistically significant for grip strength (SMD 0.57, 95 % CI 0.42 to 0.73, P < 0.00001, I^2^ = 84 %) and five chair stand time (SMD -0.56, 95 % CI -0.85 to -0.28, P < 0.0001, I^2^ = 21 %). The sensitivity analysis of grip strength indicated that some trials [18, 32] might be possible sources of heterogeneity, the degree of heterogeneity was obviously decreased by excluding them (SMD 0.37, 95 % CI 0.21 to 0.54 P < 0.00001, I^2^ = 37 %).

**Figure 5.**
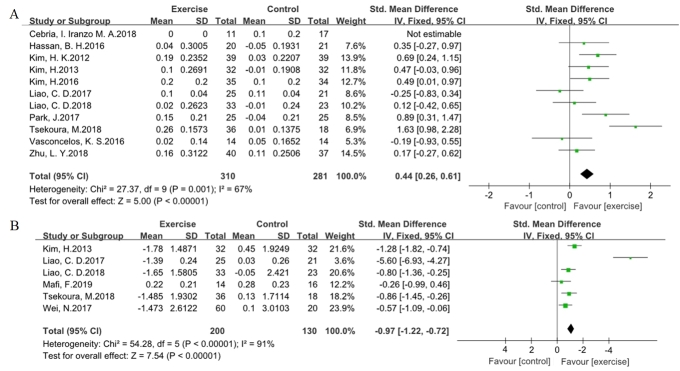
**Effects of exercise programs on the physical performance in older adults with sarcopenia**. Forest plot of difference in mean change from baseline in gait speed **(A)** and TUG test **(B)** after the intervention. CI confidence interval, IV, inverse variance, Std, standardized.

The subgroup analysis demonstrated that the association between exercise and grip strength was independent of different exercise programs (Fig. 4). Grip strength was significantly improved by resistance training (SMD 0.64, 95 % CI 0.46 to 0.83, P < 0.00001, I^2^ = 87 %), weight training (SMD 0.30, 95 % CI 0.09 to 0.51, P = 0.005, I^2^ = 12 %), and aerobic training (SMD 0.48, 95 % CI 0.13 to 0.83, P = 0.007, I^2^ = 83 %). With regard to the subtests, the I^2^ for the weight training program decreased substantially compared with the others.

### The effect on physical performance

Gait speed and the TUG test were selected to evaluate the efficacy of the exercise program on physical performance in older adults with sarcopenia (Fig. 5). Eleven trials included information for gait speed, and six trials included information for the TUG test, which were pooled together with the method of inverse variance using a fixed-effect model. The value for the change in overall effect size in the general assessment indicated that the efficacy of exercise was statistically significant for gait speed (SMD 0.44, 95 % CI 0.26 to 0.61, P < 0.00001, I^2^ = 67 %) and the TUG test (SMD -0.97, 95 % CI -1.22 to -0.72, P < 0.00001, I^2^ = 91 %). The sensitivity analysis indicated that Tsekoura’ trial [34] and Liao CD’s trial [32] might be the possible sources of heterogeneity for gait speed and the TUG test, respectively. The degree of heterogeneity was decreased by excluding the relevant trial for gait speed (SMD 0.35, 95 % CI 0.17 to 0.52 P = 0.0001, I^2^ = 40 %) and the TUG test (SMD -0.79, 95 % CI -1.05 to -0.54 P < 0.00001, I^2^ = 33 %).

### Study quality

**Figure 6.**
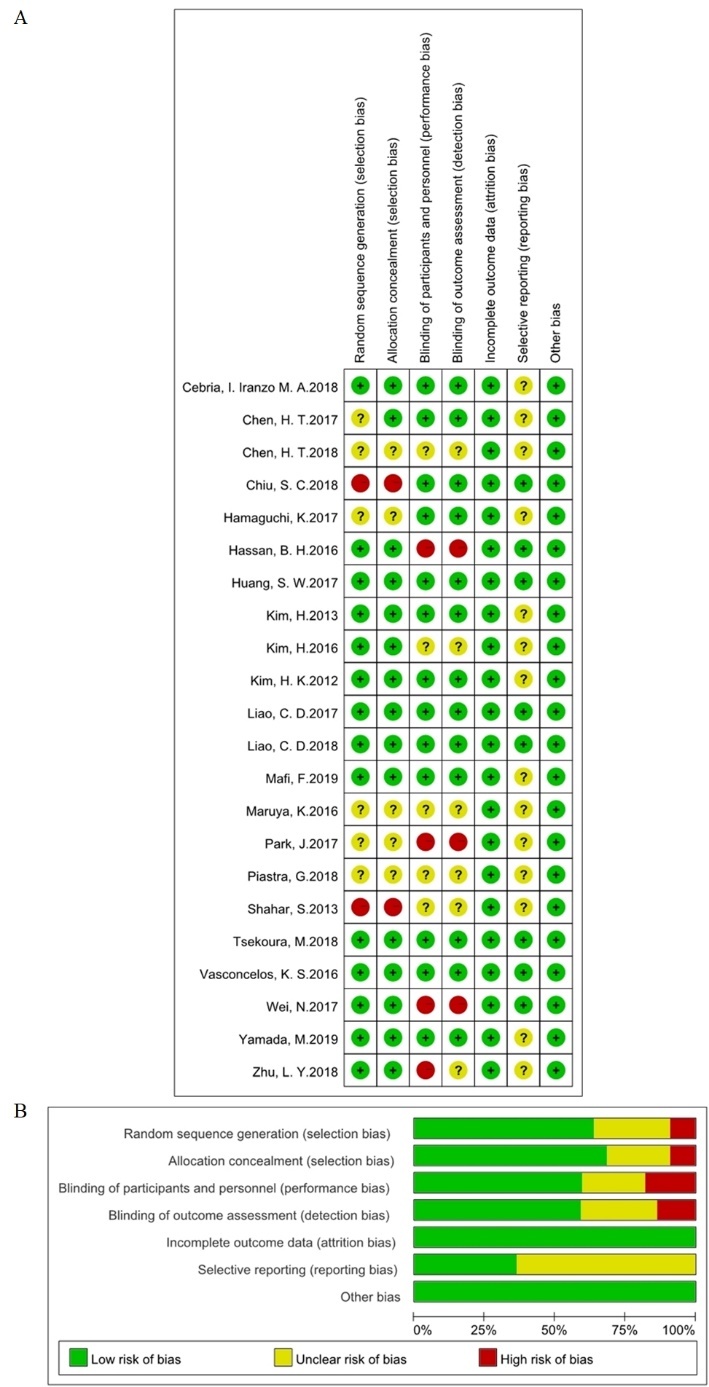
**Assessment of risk of bias based on the Cochrane risk-of-bias tool**. **(A)** Risk of bias graph; **(B)** risk of bias summary.

Details about the risks of bias of the included studies are shown in Figure 6A and Figure 6B. Four studies used single-blinded assessments, which may lead to high risks of selection bias. Two studies used nonrandomized designs, which may lead to high risks of performance and detection bias.

### Publication bias

No obvious asymmetry (Supplementary Fig. 1) from the funnel plots and no statistically significant publication bias from Egger’s regression test were observed for ASM (P = 0.718), ASM/height^2^ (P = 0.292), grip strength (P = 0.137), five chair stand time (P = 0.221), gait speed (P = 0.929) or the TUG test (P = 0.056).

## DISCUSSION

In this systematic review and meta-analysis, existing evidence from 22 randomized controlled trials and controlled clinical trials demonstrated that any type of exercises (e.g., resistance training, aerobic training, balance training, weight training, and whole-body vibration training) significantly improved muscle strength and physical performance in older adults with sarcopenia. However, the outcome of muscle mass showed no differences after exercise intervention, which is in accordance with previous studies suggesting that loss of muscle and bone mass may not be prevented by exercise [39]. In the context of the previous studies, the combination of exercise intervention and nutrition supplementation could achieve the greatest improvement in muscle mass and strength [40]. For a relevant normative result, muscle mass could be adjusted to the body size, such as the height squared, the weight or the BMI. In the present study, no differences in ASM, SMM or the muscle mass adjusted for height squared were observed between the exercise training and control groups. Low muscle mass and muscle strength are characteristic features in the definition of sarcopenia, while muscle strength is affected more than muscle mass in individuals with sarcopenia and was formerly considered the most reliable measurement [7].

Based on previous studies, any type of exercise interventions or combinations of interventions have been shown to be effective methods to treat muscle loss and weakness [41]. Currently, resistance training and aerobic training are the most common exercise programs to maintain and improve physical function in older adults [42]; while aerobic exercise is aimed at improving cardiovascular adaptations with increased peak oxygen consumption, resistance exercise is aimed at improving neuromuscular adaptations with increased muscle strength. In addition, weight training serves as an alternative to resistance training and aerobic training, it is good for balance performance and muscular coordination. Villareal, D. T. suggested that a combined exercise program provided greater improvement and prevented more adverse effects than a single exercise training program among elderly individuals [43]. Center-based and home-based exercise training are two program settings that depend on the experimental location; the former represents an informal, flexible program and is recommended for short-term interventions, while the latter represents a formal, controllable program and is recommended for long-term interventions [44].

Other reviews have reported that exercise training is generally effective for muscle strength and performance of healthy elderly adults regardless of training programs [45-47], while large clinical trials of exercise for individuals diagnosed with sarcopenia are still lacking. Compared with previous studies, our study has three strengths. First, the inclusion criteria in this meta-analysis were relatively rigid; we included only older individuals with a definite diagnosis of sarcopenia. Second, 1041 participants were enrolled in the present meta-analysis, which is twice as large as the previous reviews of sarcopenia treated by exercise [48]. Finally, we provided an integrated overview (three aspects with six outcomes) to evaluate the general effectiveness of exercise programs.

The effects of exercise programs in older adults with sarcopenia were explored in this systematic review and meta-analysis. Considerable heterogeneity (I^2^ > 50%) was inevitably detected in most of the included studies due to the complex characteristics of the exercise programs, however, there were insufficient data to conduct subgroup analyses. When excluding some trials, the degree of heterogeneity was markedly decreased. Therefore, some of the results should be interpreted with caution, and more research is needed to confirm the findings. Other important limitations of the included articles were the limited sample size, the different diagnostic criteria and the detection instruments used to diagnose sarcopenia, which may result in high heterogeneity.

In conclusion, this meta-analysis indicates that exercise programs have potential to support muscle function in elderly individuals with sarcopenia, which was recommended in the daily life. Compared with muscle mass, muscle strength and physical performance can be improved to a greater extent by exercise training. Although most of the studies suggested that regular exercise interventions improve overall performance in sarcopenic participants, more studies focused on multiple training variables and outcome measurements based on a larger population are needed to design the optimal training strategy and guide clinical practice.

## Supplementary Materials

The Supplemenantry data can be found online at: www.aginganddisease.org/EN/10.14336/AD.2019.1012.


