# Supplementary material for: Exercise Programs for Muscle Mass, Muscle Strength and Physical Performance in Older Adults with Sarcopenia: A Systematic Review and Meta-Analysis
Source: Aging Dis. 2020 Jul 23;11(4):863–73. doi: 10.14336/AD.2019.1012 (PMC7390512; doi:10.14336/AD.2019.1012)
Supplement: Supplementary file 1 [file AD-11-4-863-suppl.pdf]

SUPPLEMENTARY DATA

Exercise Programs for Muscle Mass, Muscle Strength and Physical Performance in Older Adults with Sarcopenia: A Systematic Review and Meta-Analysis

Wangxiao Bao, Yun Sun, Tianfang Zhang, Liliang Zou, Xiaohong Wu, Daming Wang, Zuobing Chen[*]

Department of Rehabilitation Medicine, First Affiliated Hospital, Zhejiang University School of Medicine, Hangzhou, China

© 2019. Bao W et al. Published online at http://www.aginganddisease.org/EN/10.14336/AD.2019.1012

SUPPLEMENTARY DATA

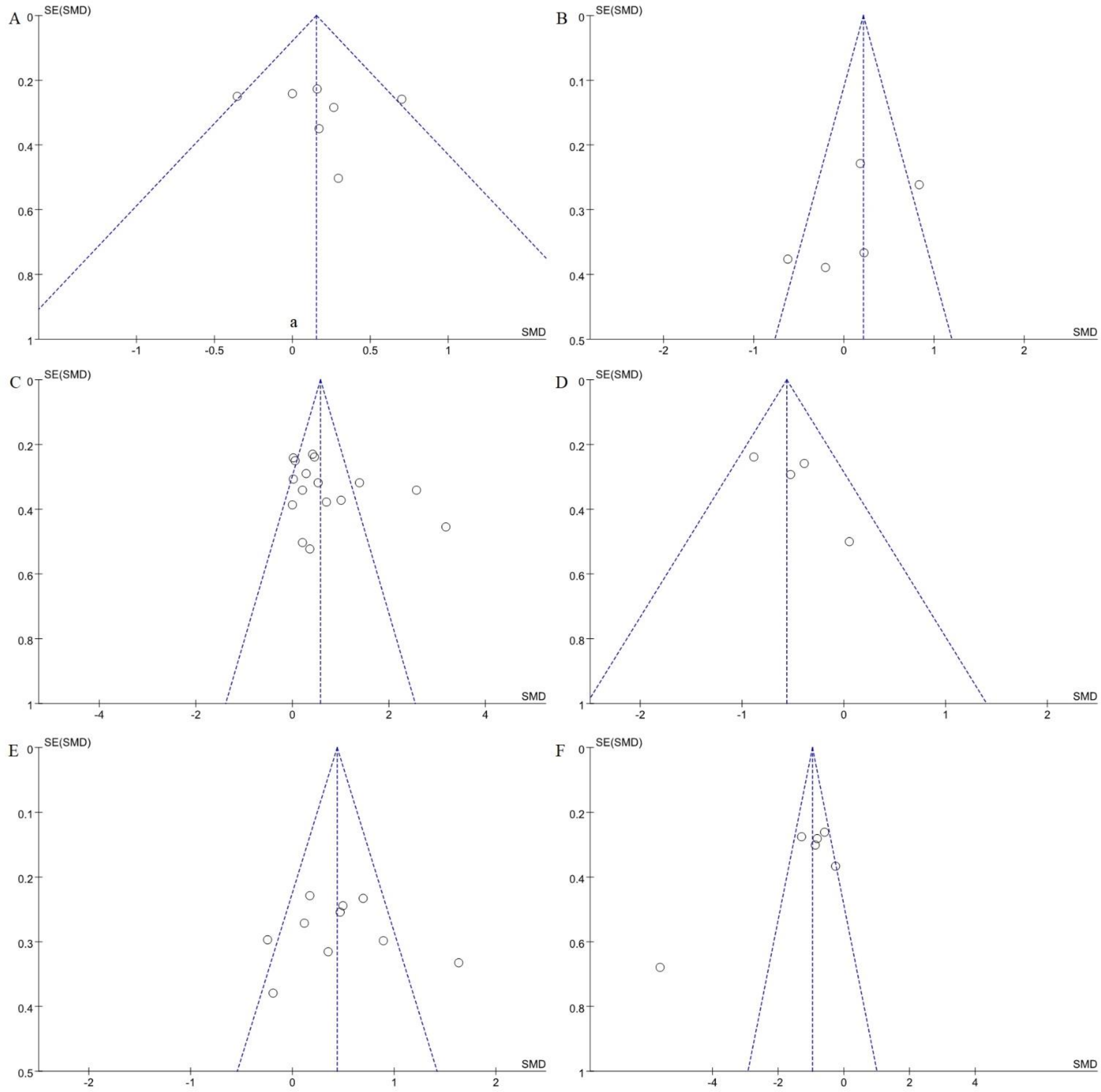

Supplementary Figure 1. Funnel Plot Asymmetry Test for publication bias analysis: ASM (A), ASM/height^2 (B), grip strength (C), five chair stands time (B), gait speed (E) and TUG test (F).

© 2019. Bao W et al. Published online at http://www.aginganddisease.org/EN/10.14336/AD.2019.1012

SUPPLEMENTARY DATA

A

Study or Subgroup	Exercise Mean	Exercise SD	Exercise Total	Control Mean	Control SD	Control Total	Weight	Std. Mean Difference IV, Fixed, 95% CI
Chen, H. T.2017	0.1	4.0509	15	-0.4	3.7	15	22.3%	0.13 [-0.59, 0.84]
Chen, H. T.2018	0.17	2.9372	17	-0.29	2.74	16	24.5%	0.16 [-0.53, 0.84]
Piastra, G.2018	1.71	6.0835	35	-0.06	6.9	37	53.2%	0.27 [-0.20, 0.73]
**Total (95% CI)**			**67**			**68**	**100.0%**	**0.21 [-0.13, 0.55]**

Heterogeneity: Chi² = 0.14, df = 2 (P = 0.93); I² = 0%
Test for overall effect: Z = 1.21 (P = 0.23)

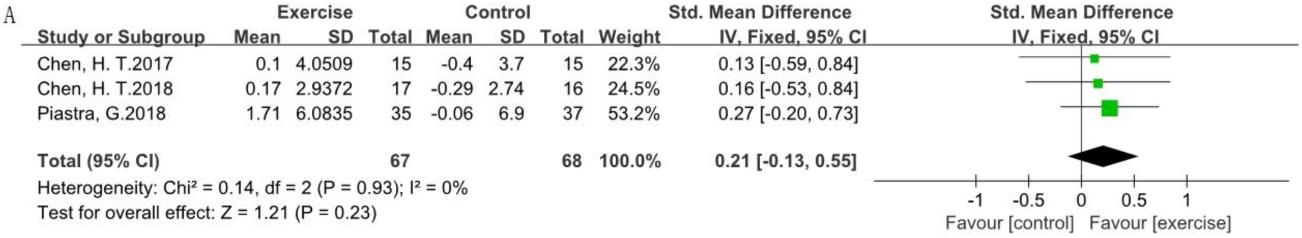

B

Study or Subgroup	Exercise Mean	Exercise SD	Exercise Total	Control Mean	Control SD	Control Total	Weight	Std. Mean Difference IV, Fixed, 95% CI
Hamaguchi, K.2017	0.1	0.3	7	0	0.46	8	8.7%	0.24 [-0.78, 1.26]
Maruya, K.2016	0.02	0.6551	26	-0.07	0.68	14	21.5%	0.13 [-0.52, 0.78]
Piastra, G.2018	0.88	2.5585	35	-0.07	2.33	37	41.7%	0.38 [-0.08, 0.85]
Tsekoura, M.2018	0.155	0.5228	36	-0.01	0.66	18	28.1%	0.28 [-0.28, 0.85]
**Total (95% CI)**			**104**			**77**	**100.0%**	**0.29 [-0.01, 0.59]**

Heterogeneity: Chi² = 0.39, df = 3 (P = 0.94); I² = 0%
Test for overall effect: Z = 1.88 (P = 0.06)

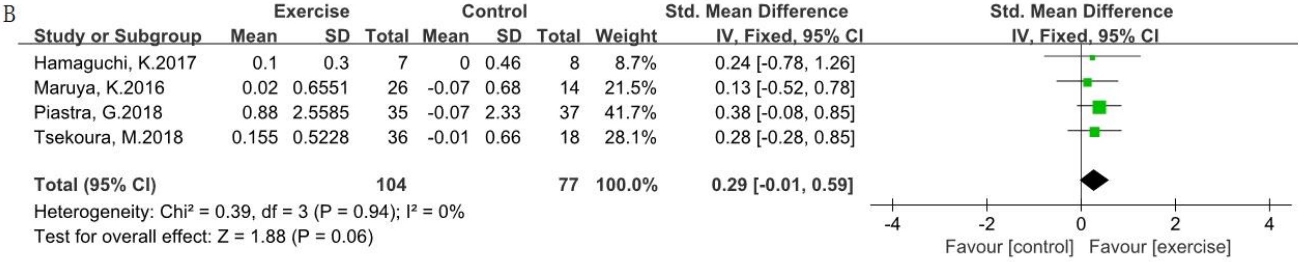

Supplementary Figure 2. Effects of exercise programs on the total body skeletal muscle mass in older adults with sarcopenia. Forest plot of difference in mean change from baseline in SMM **(A)** and SMM/height^2 **(B)** after the intervention. SMM, total body skeletal muscle mass. CI confidence interval, IV, inverse variance, Std, standardized.

© 2019. Bao W et al. Published online at http://www.aginganddisease.org/EN/10.14336/AD.2019.1012
